# Assessment of rice genotypes through the lens of morpho-physiological and biochemical traits in response to arsenic stress

**DOI:** 10.1016/j.heliyon.2024.e36093

**Published:** 2024-08-10

**Authors:** Sanaullah Jalil, Muhammad Mudassir Nazir, Mohamed A. Eweda, Faisal Zulfiqar, Hayssam M. Ali, Jean Wan Hong Yong, Xiaoli Jin

**Affiliations:** a The Advanced Seed Institute, Zhejiang Key Laboratory of Crop Germplasm, Zhejiang University, Hangzhou 310058, China; b School of Environment and Safety Engineering, Jiangsu University, Zhenjiang, 212013, China; c Department of Horticultural Sciences, Faculty of Agriculture and Environment, The Islamia University of Bahawalpur, Bahawalpur, 63100, Pakistan; d Department of Botany and Microbiology, College of Science, King Saud University, Riyadh, 11451, Saudi Arabia; e Department of Biosystems and Technology, Swedish University of Agricultural Sciences, 23456, Alnarp, Sweden

**Keywords:** Antioxidants, Arsenic, Catalase, Malondialdehyde, Rice, ROS

## Abstract

Rice is a globally important food crop which is sensitive to the presence of a metalloid, arsenic (As). There is limited research pertaining to identifying relevant As-tolerant rice germplasm in adaptive breeding research initiatives, despite the fact that As contamination in rice has long been known. This study served to identify the growth performance of different rice genotypes under high levels of As. Rice seed germination analysis (germination percentage, GP) was performed to categorize the eight different rice genotypes and growing under varying As levels including As_25_, 25 μM and As_50_, 50 μM. The Zhenong 41 was identified as the highly tolerant genotypes with lowest decrease in GP by 87 %, plant height (PH) by 26 %, and dry weight (DW) by 16 %; while 9311 was observed to be the most sensitive genotype with highest reduction in GP by 44 %, PH by 48 % and DW by 54 % under As_25_ stress conditions, compared to control treatment. The higher As_50_ stress treatment delivered more adverse growth inhibitory effects than the rice plants cultivated under As_25_. Specifically, the As-sensitive rice genotype 9311 showed significantly higher decrease in foliar chlorophyll contents relative to the other genotypes, especially Zhenong 41 (As-tolerant). During exposure to high As levels, the rice genotype 9311 significantly modulated and augmented the production of MDA and H_2_O_2_ by stimulating the activities of POD, SOD, and CAT. This study revealed interesting insights into the responses of rice genotypes to variable As stresses throughout the various growth stages. Overall, the findings of this study could be harnessed to support any ongoing As-tolerant rice breeding agendas for cultivation in As-polluted environments.

## 1 Introduction

Rice, is the most extensively consumed grains worldwide, functions as a dietary staple, particularly in Asian countries where it is a fundamental component of daily meals [1]. It serves as a pivotal source of carbohydrates, energy, and vital nutrients, nourishing billions of people every day [2]. Therefore, in anticipation of Asia's projected population growth by 2050, a substantial surge of 60 %–70 % in rice production is imperative to meet the escalating demands, grain supply reliability, grain safety, and with minimal impact to the environment [[3], [69], [70], [71]]. Heavy metals and metalloids are known environmental pollutants, and their toxicity is of increasing significance for agricultural, ecological, evolutionary, nutritional and environmental reasons [[56], [57], [58], [59], [60], [61], [62], [63], [64], [65]]. Despite the nutritional significance and extensive utilization of rice, arsenic (As) contamination has emerged as recent alarming issue [[4], [70], [71]]. The element Arsenic (As) is a poisonous metal/metalloid that is widely recognized as a carcinogen affecting humans [[5], [55],]. Anthropogenic activities such as sewage sludge, industrial waste, mining and the use of As-containing agrochemicals contribute to As contamination in rice-growing regions. The anthropogenic sources exacerbate the problem, leading to enhanced accumulation levels of As in rice grains [[6], [70], [71]]. Moreover, exposure to As-contaminated rice poses various health issues to individuals, including: cardiovascular diseases, leukaemia, skin lesions, diabetes, lung cancer, kidney cancer, and reproductive abnormalities like premature birth [7]. In addition, many rice growing areas have very high levels of As in the groundwater; the WHO's recommended level of As was established at 10 ppb (parts per billion) for drinking water [[8], [55]]. However, due to the lack of policy guidelines and environmental chemistry technical limitations in many developing countries, there is no formal guidelines for As levels in irrigation water. To lower the levels of As in grains and straws below the safe levels for ingestion by humans and animals, one of the plausible option was to develop As tolerant rice varieties/genotypes. The ill effects of As have been extensively documented, revealing its ability to significantly impede metabolic functions, hinder development, and inhibit growth in plants [6,7]. Rice, barley, maize and wheat grains serves as primary source of As ingestion for humans. As major part of the population relies on rice food, its higher tendency to accumulate As, especially in the grains, makes it a major contributor to the incorporation of inorganic As into the human food system compared to other grain crops [8]. By 2050, the population is anticipated to steadily increase to 9.7 billion people, creating a pressing need for a 50 % increase in crop yield production compared to current levels [[5], [6], [7]]. Rice, as the essential staple food worldwide, is a major contributor in the livelihoods of millions of people, particularly in Asian nations, where it constitutes 90 % of both production and consumption. Specifically, the As accumulation in paddy soils and rice cultivation in polluted waters will ultimately jeopardize the health of people and animals.

The presence manifestation of As in the irrigation water and topsoil cause diverse hazardous impacts on rice plant, often compounded by additional soil-related challenges inherent to rice cultivation and such problems frequently congregated with other soil-related issues in rice farming [9]. The occurrence of As in rice grown fields triggers several detrimental responses, such as deprived germination, poor roots establishment, reduced photosynthesis activity, decreased biomass, leaf discoloration and stunted plant growth [10]. The impact of As toxicity on rice plants depends on its chemical structure [11]. While, the incorporation and translocation of inorganic As species are highly relatively effective into rice plant system [12]. Among the inorganic As sources, the more hazardous trivalent arsenite AsIII and the less poisonous pentavalent arsenate AsV are the main species found in water and soils [13]. Arsenite AsIII dominates in anaerobic lowland submerged paddy soils, while arsenate AsV prevails in aerobic rainfed upland paddy soils [14,15]. The strong affinity of AsIII(III) for sulfhydryl-containing enzymes negatively affects the functionality of important enzymes in plants [16]. Despite the apparent scrutiny on the interaction between rice and As for the past two decades, there remains limited knowledge about the most As-resistant cultivars, genotypic variants, and screening techniques. At present, there is no known As-excluding donor germplasm; or useful As-related germplasm utilized in breeding programmes to develop As-resistant rice cultivars. In regions heavily affected by As contamination, many farmers face extreme poverty and lack the resources to afford expensive mitigation techniques or alternative food sources [16]. Furthermore, exposure to As stress leads to phytotoxic effects on rice plants through escalating electrolyte leakage, and membrane damage levels [17].

During an exposure to high As level by plants, there will be a biochemical burst of reactive oxygen species (ROS); consequently supressing the antioxidant defence system. To counteract the effects of ROS during high As exposure, rice plants will employ the antioxidant defence mechanisms to cope with the pertubation induced by the ROS [18]. In addition, various antioxidative components also contribute to the neutralization of ROS generated under As toxicity [19]. Under stress scenarios, the antioxidant enzymes activities repressed due to the imbalances in enzyme functioning, the glyoxalase system and biomolecules. This biochemical imbalance within the tissues ultimately results in excessive oxidative stress [20]. Despite significant attention on the interaction between rice and As in the past two decades, there remains limited knowledge about the most resistant cultivars, genotypic variants, and screening techniques. At present, there is no known As-excluding donor germplasm has been identified or utilized in breeding efforts to develop resistant rice cultivars. In regions heavily affected by As contamination, many farmers face environmental challenges, extreme poverty and limited resources to cope with As-related pollution [21].

We selected and assessed the eight rice genotypes growing under different levels of As stress in order to comprehend the variation in genetic makeup concerning germination ability, alteration in photosynthetic parameters, growth related traits, and the modulation of their antioxidant and ROS system. Based on our hypothesis, the selected rice genotypes were expected to exhibit a wide range of morphological trait diversity under As-induced toxicity. The objectives were as follows: (a) to determine the germination potential of selected genotypes under various levels of As stress; (b) to investigate the genetic susceptibility/tolerance behaviour of selected rice genotypes against As toxicity; and (c) to identify the most significant As-sensitive genotype. The identified As-sensitive genotype could be used for future research pertaining to developing mitigation strategies against high levels of As.

## 2 Plant materials and methods

### 2.1 Plant materials and study layout

Eight different rice genotypes: *Oryza sativa* subsp. japonica(Nipponbare); 7 *Oryza sativa* subsp. indica rice (9311, Guiyin 206, Minghui 63, Zhenong 34, Zhenong 41, Zhenong 37 and Shenghai 1), were selected to assess their responses to As-induced toxicity. During the year of 2023, the genotypes were carefully selected and the whole experiment was conducted using a Randomized Complete Block Design (RCBD) at the Jin's laboratory, College of Agriculture and Biotechnology, Zhejiang University, Hangzhou, China; three replication of each treatment for the entire study.

### 2.2 Seed germination analysis

Seed germination analysis was done at 25 °C in a growth chamber to observe the germination ability of all selected genotypes under varied levels of As stress. Surface sterilize the rice seeds by soaking them in a 2 % H_2_O_2_ (v/v) solution for 30 min. Seed rinsing was again done with distilled water. The seeds of each genotype were germinated in hydroponic growth medium within the boxes of 12 cm × 18 cm size; consisted 30 seeds of each genotype. Three treatments of inorganic As, 0 μM (control, CK), 25 μM (As_25_) and 50 μM (As_50_) were applied in the form of sodium arsenate dibasic heptahydrate (AsH_15_Na_2_O_11_) to determine the germination ability. Three replicates were prepared for each genotype and the respective As concentrations, in order to ensure statistical validity. After 10 days of seed sowing, the number of germinated seeds was noted. The germination percentage was evaluated by dividing the germinated seeds numbers by the total sowed seeds and multiplying by 100, in all treatments. After examining the germination percentages, the genotypes were organised into four groups including: highly tolerant >80 %, moderately tolerant >50 %, moderately susceptible <50 %; and the highly susceptible <20 %; following the formula of Murugaiyan et al. [22] for the As tolerance index (germination %).

### 2.3 Plant growth conditions for seedling stage screening

The germinated seeds of all the selected genotypes were subsequently transferred and grown into plastic pots under hydroponic conditions to evaluate the response of genotypes under different As concentrations (0, 25 and 50 μM ) at seedling stage of rice plants. The pots were then adjusted in a growth chamber having 8 h dark and 16 h light photoperiod along with 22 °C for dark photoperiod and 28 °C for light photoperiod, light intensity (250 μmol, m^−2^ s^−1^) and relative humidity of 70 %. During the preliminary 7 d after transplantation, the pots were irrigated with half-strength hydroponic nutrient solution (HNS). Following which, they were switched to full-strength HNS for the next 14 d during the experiment [11].

### 2.4 SPAD value and plant growth analysis

The relative foliar chlorophyll contents (SPAD values) were noted with a portable instrument (SPAD-502^+^, Tokyo, Japan) 7 d after As treatment [67]. Plant sample collection for the other analysis was also done on the same day. The roots of seedlings were washed with double distilled water (ddH_2_O). Plant height (Ph) was measured with a ruler while an analytical balance was employed to determine the dry weight of rice seedlings following Jalil et al. [11]. Furthermore, a subgroup of these samples was frozen using liquid nitrogen and then stored at −80 ℃ [23,24].

### 2.5 Determination of arsenic contents

The roots of rice seedling were cleaned with ddH_2_O. Then they were soaked in EDTA (20 mM) at 25 °C for 30 min in order to remove adhered metal ions on the root surface. Later, the root samples were oven-dried at 70 °C for 48 h. The digestion of the dried samples was done following the Nazir et al. [24]. After digestion, the samples were diluted in ddH_2_O and final volume (10 mL) was made. For As contents quantification, the digested samples were in an atomic absorption spectrophotometer (AA6300, Shimadzu, Japan) [25].

### 2.6 Measurement of malondialdehyde and hydrogen peroxide contents

The malondialdehyde (MDA) contents from the shoot samples were determined following the methods of Morales and Munné-Bosch [26] and while H_2_O_2_ contents were determined by described methods of Srivastava et al. [27]. The details of protocols have been presented in Jalil et al. [11] and with some modifications [68].

### 2.7 Measurement of antioxidant enzyme activities

The sample (0.5 g) from shoots was taken and the extracts were made following the protocols detailed described in our previous study by Jalil et al. [11]. SOD enzyme activity was measured at 560 nm following the protocol of Giannopolitis and Ries [28]. POD activity was measured following the methodology of Maehly [29] at 470 nm. Moreover, CAT activity was recorded at 240 nm following Aebi [30].

### 2.8 Statistical analysis

Statistical analysis was undertaken using Statistix 10. The data on the graphs were shown as the mean and standard error (SE) of three replicates; these were plotted later using Graph Pad Prism (8.0.2). Data were exposed to two way analysis of variance and significant means were divided using LSD at 5 % level of probability.

## 3 Results

### 3.1 Effects of arsenic on seed germination

**Fig. 1:**
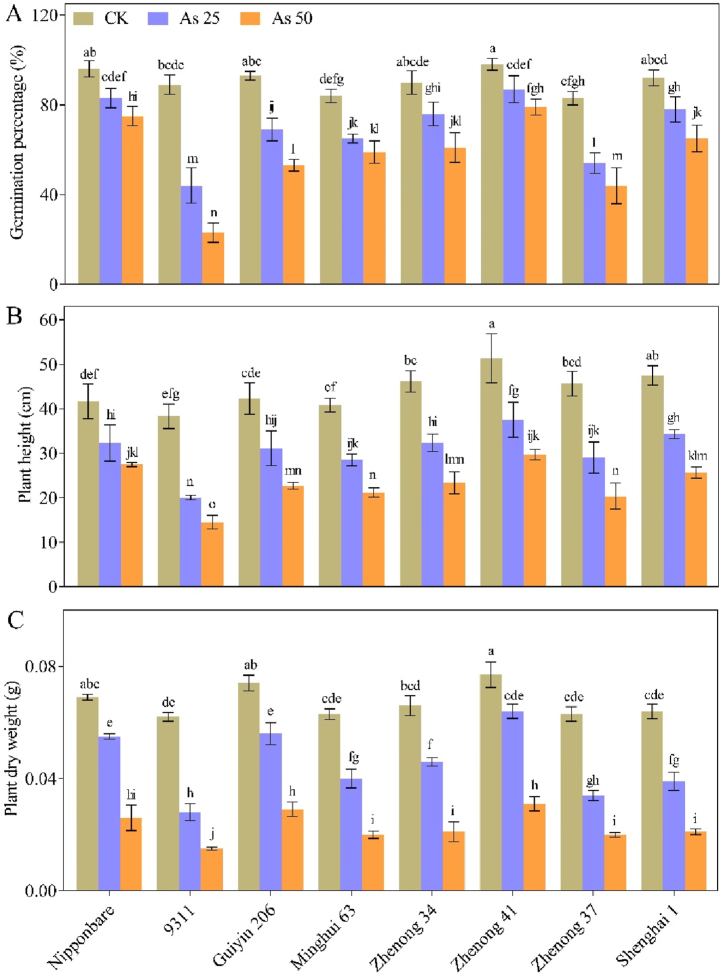
Effects of arsenic (As) treatments on the (**A**) seed germination and (**BC**) plant growth traits of various rice genotypes. Vertical bars depict the mean ± SD of three replicates. Variation in the above bar letters highlights significant difference at *p* ≤ 0.05.” Treatment details include: As_50_ as As 50 μM L^−1^; As_25_ as As 25 μM L^−1^; and CK as control.

The different rice genotypes response varied under altered As stress conditions, significantly affecting their germination capacity. When the concentrations of As were increased, the germination percentages (GP) of the genotypes showed a marked reduction. Under control conditions (without As), all eight genotypes demonstrated a high GP, with the Zhenong 41 genotype exhibiting the highest germination rate of 98 %. However, the impact of varying As concentrations on GP revealed more pronounced effects across the genotypes (Fig. 1A). However, the better response of genotypes to As stress was showed under the As_25_ treatment compared with the As_50_ treatment, as observed in seed germination analysis. Specifically, the results of As_25_ treatment demonstrated that Nipponbare and Zhenong 41 genotypes exhibited germination rates of 83 % and 87 %, respectively, which is more than 80 % and categorized as highly tolerant genotypes. Conversely, the genotype 9311 displayed a GP of 44 % and was classified as moderately susceptible genotype. Furthermore, the Guiyin 206, Minghui 63, Zhenong 34, Zhenong 37 and Shenghai 1 genotypes exhibited GP ranging between 50 and 80 %, placing them in moderately tolerant category. Interestingly, all genotypes represented a substantial decline in the GP under As_50_ treatment, even the tolerant genotypes (Nipponbare and Zhenong 41), which showed moderate tolerance against the As stress. Specifically, Zhenong 37 was re-classified from the moderately tolerant category to the moderately susceptible category with a GP of 41 %. These findings indicated that the As_50_ treatment was more hazardous for plants of all genotypes than the As_25_ treatment.

### 3.2 Plant growth parameters

The exposure of all eight genotypes to different concentrations of As for a duration of 21 days resulted in the substantial reduction in plant height (PH) and dry weight (DW), compared with the plants grown under control conditions. The genotype 9311 exhibited the most substantial reduction, with a decrease of 48 % and 62 % in PH, and 54 % and 75 % in DW under the As_25_ and As_50_ treatments, respectively, than control (CK). In contrast, the least significant decrease in PH by 22 % and 34 % was observed in Nipponbare, while substantial reduction in DW by 16 % and 60 %, respectively was observed in Zhenong 41 under As_25_ and As_50_ treatments, compared with CK treatment (Fig. 1B and C). Other genotypes also displayed variable responses to growth parameters with suppression of PH (ranging from 26 % to 36 %) and DW (ranging from 20 % to 40 %) under As_25_ stress conditions. Overall, our findings indicated that the As_50_ treatment delivered more negative effects on the growth parameters compared to the As_25_ treatment.

### 3.3 Chlorophyll content and As contents

**Fig. 2:**
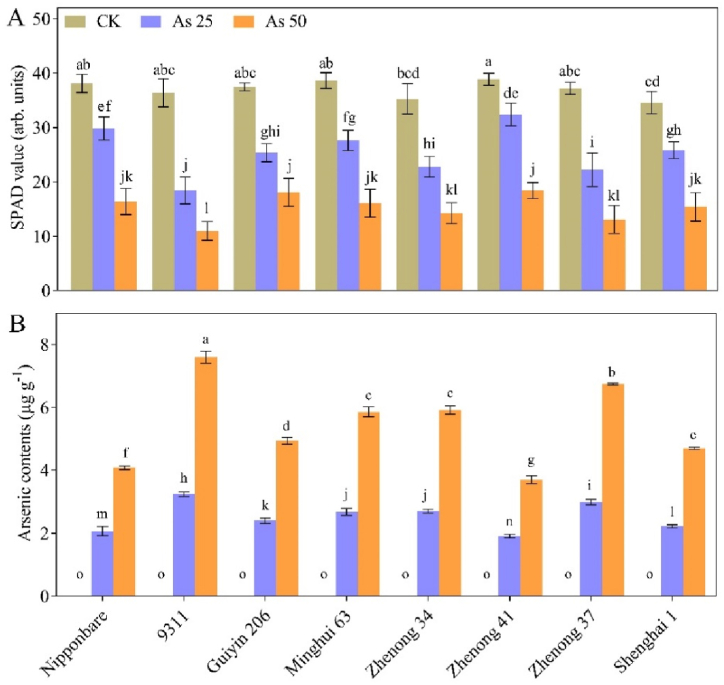
Effects of arsenic (As) treatments on the (**A**) SPAD value and (**B**) As contents of various rice genotypes. Vertical bars depict the mean + SD of three replicates. Variation in the above bar letters highlights significant difference at p ≤ 0.05. Treatment details include: As50 as As 50 μM L^−1^; As25 as As 25 μM L^−1^; and CK as control.

We observed a reduction in the leaf SPAD value due to As stress, however, no significant differences were found among genotypes under control conditions. The genotype 9311 displayed the most significant reduction in SPAD values, experiencing a substantial decrease of 49 % and 70 % under the As_25_ and As_50_ treatments, respectively, compared to the CK treatment. Following closely, genotype Zhenong 37 exhibited reductions of 40 % and 65 % under the same and As_50_ treatments, respectively. (Fig. 2A), indicating their high sensitivity to As stress. Other genotypes showed modest reductions in SPAD value compared to mentioned genotypes. For instance, Zhenong 41 showed a minimal decrease of 17 % in SPAD value under As_25_ treatment. However, under As_50_ stress conditions, there was a more pronounced toxic effect on SPAD values compared to the As_25_ stress conditions. Furthermore, we determined the accumulation of As in plants of all genotypes under As_25_ and As_50_ treatments. The results revealed that genotypes under As_50_ treatment contained higher concentration of As compared to those under As_25_ treatment (Fig. 2B). In addition, among all genotypes, 9311 accumulated a substantially higher amount of As, while Zhenong 41 accumulated a lower amount of As compared to other genotypes, indicating their sensitivity and tolerance, respectively to As stress conditions.

### 3.4 Quantification of MDA and H_2_O_2_ contents

**Fig. 3:**
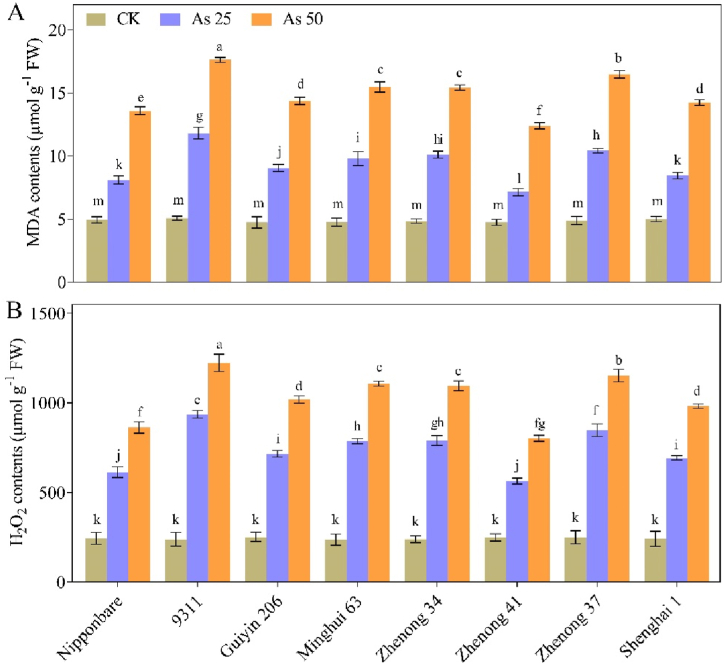
Effects of arsenic (As) treatments on the (A) MDA (malondialdehyde), and (B) H_2_O_2_ (hydrogen peroxide) content of various rice genotypes. Vertical bars depict the mean ± SD of three replicates. Variation in the above bar letters highlights significant difference at *p* ≤ 0.05.” Treatment details include: As_50_ as As 50 μM L^−1^; As_25_ as As 25 μM L^−1^; and CK as control.

The values of MDA contents and H_2_O_2_ production did not differ significantly from each other (Fig. 3A and B). However, when exposed to As stress, a considerable upsurge was observed in the values of MDA contents by 39 %, 57 %, 48 %, 51 %, 52 %, 34 %, 53 % and 41 % and H_2_O_2_ contents by 60 %, 75 %, 65 %, 70 %, 69 %, 56 %, 71 % and 65 %, in Nipponbare, 9311, Guiyin 206, Minghui 63, Zhenong 34, Zhenong 41, Zhenong 37 and Shenghai 1, respectively under the As_25_ treatment. Similar trend was observed under As_50_ treatment, but with a more substantial increase in values compared with As_25_ treatment (Fig. 3A and B). Our results demonstrated that the values of MDA and H_2_O_2_ significantly enhanced in the As-sensitive genotypes, while only slight increases were observed in the values of As tolerant genotypes, particularly in more sensitive genotype 9311 and more tolerant genotype Zhenong 41.

### 3.5 Antioxidant enzymes assays

**Fig. 4:**
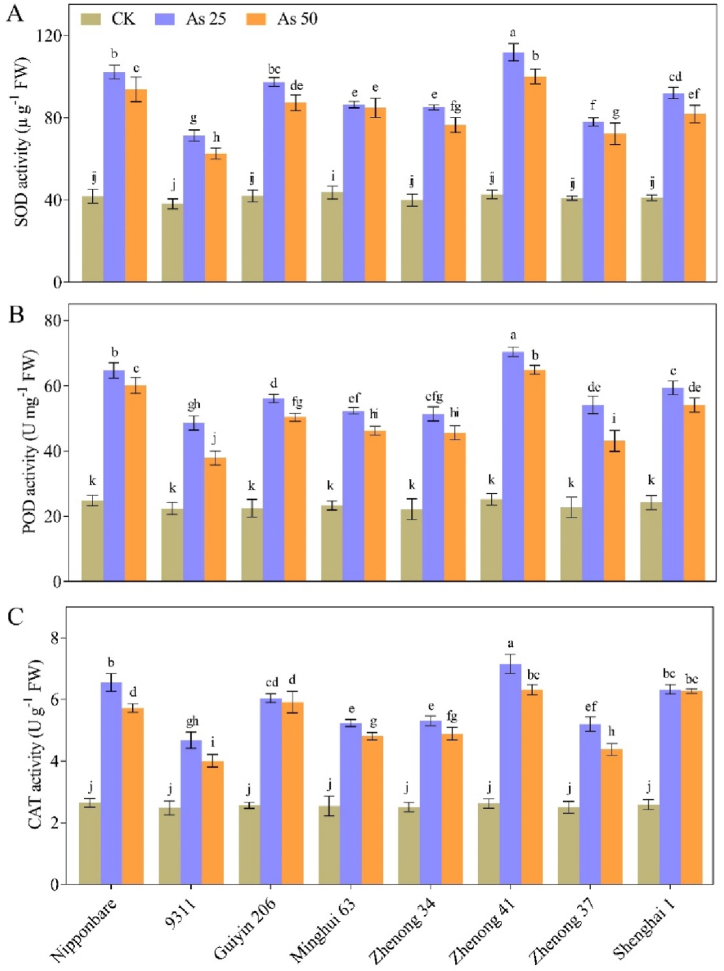
Effects of arsenic (As) treatments on the (**A**) SOD (superoxide dismutase), (**B**) POD (peroxidase) and (**C**) CAT (catalase) activity of various rice genotypes. Vertical bars depict the mean ± SD of three replicates. Variation in the above bar letters highlights significant difference at *p* ≤ 0.05. Treatment details include: As_50_ as As 50 μM L^−1^; As_25_ as As 25 μM L^−1^; and CK as control.

All genotypes increased their antioxidant levels under As stress. Interestingly, these increases were more obvious in the values of SOD, POD and CAT activities within the high As tolerant rice genotype, Zhenong 41 with increase of 60 %, 66 % and 63 %, respectively under As_25_ stress conditions as compared with CK plants (Fig. 4A,B,C). In contrast, the SOD, POD and CAT activities in the As-sensitive rice genotype, 9311, were significantly lower, with increase of 47 %, 54 % and 45 %, respectively, compared with CK treatment. The other genotypes showed modest increase in the levels of antioxidant enzyme activities; and represented non-significant differences among each other. Additionally, all the genotypes showed similar trend under As_50_ stress conditions but with slightly reduction in SOD, POD and CAT values, comparison with As_25_ stress conditions. Moreover, no noteworthy change was found in enzyme activities in plants from all genotypes grown under the controlled conditions without As stress.

## 4 Discussion

Among all abiotic stress factors, the potential hazards posed by heavy metals and metalloids (HMs) are one of the main concerns regarding human and environmental health issues [31,[32], [70], [71]]. HMs have high reactivity due to their oxidative state and caused consequent toxicity to the majority of species [33]. The pollution caused by HMs has significantly impeded crop production in recent years. In addition, HMs severely jeopardizes the human food and nutrition, especially when it comes to rice [34]. The As is considered to be one of the most harmful HM and recognized as non-essential and hazardous HM due to its no essential physiological roles in plants [[35], [56], [71]]. The enhanced accumulation of As disturbs the plant development and growth, leading to substantial changes in the morphological and physiological functions of plants; specifically, the high As exposure in rice will cause the following: lower seed germination, poor seedling health, reduced photosynthesis, reduced biomass, and negative alteration of metabolic activities and ROS production. The toxicity of As also caused strait head disease in rice grown in soils with high As levels [10,17]. It is of utmost importance to take the appropriate action to address the issue of As toxicity in rice. Several research reports have explored the hazardous impacts of As toxicity in rice [[27], [36],^37^]. In line with these concerns, the findings revealed a reduction in seed germination and overall plant growth under As stress. The application of two different concentration of As_25_ and As_50_ led to a decrease in GP, DW and PH of rice seedlings (Fig. 1), corroborating the results of Murugaiyan et al. [22] and Kumar et al. [38]. Based on the GP ratios, we classified the studied genotypes into different As tolerance groups. Genotype 9311 was identified as a moderately susceptible genotype (GP <50 %), while Nipponbare and Zhenong 41 with GP exceeding 80 %, were classified as highly tolerant genotypes. The remaining five genotypes exhibited moderate tolerance under the As_25_ stress conditions (Fig. 1). Furthermore, it was observed that As_50_ treatment resulted in more pronounced deleterious effects on GP of genotypes, indicating that higher concentrations of As severely impaired the growth of rice plants. This study aligned with previous research representing the detrimental effects of As stress on plant phenotypic parameters in crops such as soybean, canola, and barley [24,39]. Additionally, the reports on Cr toxicity in rice crops have also reported negative impacts on seed germination rates [[40], [65]], which parallel the results obtained in our study under As toxicity.

Chlorophyll, an essential component of plants involved in the photosynthesis and an integral component in plant development and serves as a valuable indicator to find stress levels caused by HMs in crop plants [[41], [65], [66]]. In our study, we lower SPAD values of rice seedlings under As stress, and these reductions in SPAD value increased with higher As concentrations (Fig. 2). The decline in chlorophyll content was correlated with a decrease in plant biomass [[42], [67]]. Consistent with previous experiments, we observed a positive link between chlorophyll contents and shoot development in rice plants. The high levels of metalloid As prevented the production of chlorophyll contents by interfering with the action of chlorophyll synthase. All the rice genotypes in our study were adversely affected by As stress, except for Zhenong 41, which exhibited high tolerance against As toxicity. In contrast, genotype 9311 showed increased susceptibility to As toxicity, resulting in more pronounced detrimental effects on SPAD value. Additionally, our findings revealed that the rice genotypes generally accumulated higher amount of As concentration under As_50_ treatment than the As_25_ treatment; and these higher As concentrations were associated with more negative effects on plant growth parameters compared to plants grown. These results highlighted the negative correlation between growth parameters and As accumulation, indicating that the rice growth parameters were suppressed in a dose-dependent manner which was consistent with the Mousavi et al. [43] and Wu et al. [44].

Antioxidants, both non-enzymatic and enzymatic, have a helpful role in the defense by working to balance ROS generated within different cellular compartments [45]. In this experiment, we observed that the exposure of rice genotypes to varying levels of As toxicity led to a burst of H_2_O_2_ and MDA contents in rice plants, particularly in the 9311 genotype, which displayed a sensitive response to As compared to other genotypes (Fig. 3). This increase in ROS production and MDA contents indicates the damage induced by As toxicity, leading to membrane degradation, lipid peroxidation, and enhanced oxidative damage to cells. These results were in agreement with the previous reports that have demonstrated similar As-induced oxidative indicators in barley, maize and wheat etc. [24,46,47]. In contrast, Zhenong 41 showed a low amount of MDA contents and H_2_O_2_ production, indicating its high tolerance towards the As phytotoxic effects. It was plausible that the Zhenong 41 genotype possessed an efficient As-detoxification system, which reduced oxidative stress and enhanced the capacity for photosynthetic activity, leading to improved growth and biomass of rice seedlings. Gupta and Ahmad [48] had previously described in their study about differential responses of various rice genotypes to As toxicity, thereby supporting our findings. Our results also aligned with the observations of Khan et al. [49], whom reported excessive lipid peroxidation and ROS formation in As-sensitive rice cultivars growth under high As levels. Thus, the inhibitory role of higher As concentrations on the biology of rice plants health could be attributed to oxidative injury to the tissues [50]. Earlier studies have also supported the notion that plants develop mechanisms to promote cellular metabolism and to eliminate the production of free radicals; subsequently lowering the ROS levels and MDA contents [50,51].

To mitigate the damaging effects of oxidative injury due to higher As levels, plants have developed a specific mechanism known as the antioxidant dependant defence process [[52], [63], [65], [68]]. Our study investigated how different rice genotypes remarkably boosted the activities of antioxidative enzymes, to combat As based oxidative injury (Fig. 4). It was observed that rice plants activated the antioxidant defence system to eliminate excessive ROS production within their cells, consequently reducing MDA levels. In our study, we observed a substantial increase in H_2_O_2_ production and MDA contents with higher concentrations of As toxicity, indicating a positive correlation between these variables and As accumulation. Several studies have also reported similar findings in rice, wheat, and maize plants, demonstrating the activation of these antioxidative indicators [47,53,54]. Higher antioxidant enzyme activities were found in the As-tolerant rice genotypes, particularly Zhenong 41, compared to the As-sensitive genotype 9311; indicating a compromised response to As stress in the latter. It is possible that the As-sensitive rice genotype fails to maintain the redox balance, leading to excessive ROS accumulation within the cellular components under As stress. Therefore, the proposed method described a plausible approach for mitigating As stress in rice plants, particularly in As-sensitive cultivars such as 9311, which could be further selected in future research.

## 5 Conclusion

The study examined the effects of As, a non-essential and toxic HM, on rice genotypes. The study revealed that high As levels reduced seed germination, SPAD value, growth, and biomass of rice seedlings. Specifically, the higher As concentrations exacerbated the negative growth effects. The study also highlighted the complex interplay between oxidative stress and As toxicity. Rice genotypes responded to high As levels by activating antioxidant defense mechanisms, as evidenced by the heightened antioxidative enzyme activities. Interestingly, the rice genotype-specific responses were evident, with Zhenong 41 demonstrating notable As tolerance through effective maintenance of redox balance and adaptations; while 9311 was considered a susceptible genotype to As stress. These insights provided by this study contributed to the broader understanding of rice responses to As toxicity and offered plausible solutions in addressing this critical environmental issue affecting food safety.

## Funding

This work was supported by the Science and Technology Office of Zhejiang Province, China (project no. 2021C02063-6). This work was further funded by the Researchers Supporting Project number (RSP2024R123), 10.13039/501100002383King Saud University, Riyadh, Saudi Arabia.

## Data availability statement

Data will be made available on request to corresponding authors.

## CRediT authorship contribution statement

**Sanaullah Jalil:** Writing – original draft, Validation, Methodology, Conceptualization. **Muhammad Mudassir Nazir:** Writing – review & editing. **Mohamed A. Eweda:** Writing – review & editing. **Faisal Zulfiqar:** Writing – review & editing, Validation, Formal analysis. **Hayssam M. Ali:** Writing – review & editing. **Jean Wan Hong Yong:** Writing – review & editing. **Xiaoli Jin:** Writing – review & editing, Supervision.

## Declaration of competing interest

The authors declared that they have no known competing financial interests or personal relationships that could have appeared to influence the work reported in this paper.
